# *In Vitro* Cytotoxicity and* In Vivo* Antimammary Tumor Effects of the Hydroethanolic Extract of* Acacia seyal* (Mimosaceae) Stem Bark

**DOI:** 10.1155/2018/2024602

**Published:** 2018-03-25

**Authors:** Stephane Zingue, Amstrong Nang Njuh, Alain Brice Tueche, Jeremie Tamsa, Edwige Nana Tchoupang, Stanislas Djaouli Kakene, Marius Trésor Kemegne Sipping, Dieudonné Njamen

**Affiliations:** ^1^Department of Life and Earth Sciences, Higher Teachers' Training College, University of Maroua, P.O. Box 55, Maroua, Cameroon; ^2^Department of Animal Biology and Physiology, Faculty of Science, University of Yaounde 1, P.O. Box 812, Yaounde, Cameroon; ^3^Department of Biological Sciences, Faculty of Science, University of Maroua, P.O. Box 814, Maroua, Cameroon; ^4^Department of Biochemistry, Faculty of Sciences, University of Yaounde 1, P.O. Box 812, Yaounde, Cameroon

## Abstract

The present study was designed to evaluate the* in vitro* and* in vivo* antitumor effects of* A. seyal* hydroethanolic extract on breast cancer. The cytotoxicity of* A. seyal *extract was evaluated using resazurin reduction assay in 9 cell lines. Further, the protective effect of the hydroethanolic extract of* A. seyal *stem barks was evaluated on 7,12-dimethylbenz(a)anthracene- (DMBA-) induced breast cancer rat model. Incidence, burden, volume, and histological analysis of mammary tumors were measured. The* Acacia seyal *extract exhibited CC_50_ of 100 in MCF-7 cells after 24 h.* In vivo*, no tumors were detected in rats from the control group, while 11 rats out of 12 (91.66%) developed mammary tumors in the DMBA-exposed group receiving only the vehicle.* Acacia seyal* extract significantly (*p* < 0.01) and in the dose-dependent manner reduced tumor incidence (3 rats out of 12 at the dose of 300 mg/kg), burden [62.1% (150 mg/kg) and 65.8% (300 mg/kg)], and mass. It protected rats against DMBA-induced breast hyperplasia, with an optimal effect at the dose of 300 mg/kg. Taken altogether, these results suggest that the hydroethanolic extract of* Acacia seyal* might contain phytoconstituents endowed with antitumoral properties, which could protect against the breast cancer induced in rats.

## 1. Introduction

Cancer is a major public health problem worldwide [1, 2]. In the year 2015, global cancer cases were estimated to 15.2 million with 8.9 million of deaths [3]. The three most commonly diagnosed cancers in women include breast, lung and bronchus, and colorectal cancer [2]. According to the same authors, in 2016, breast cancer alone is expected to account for 29% of all new cancers diagnosed in women. In Cameroon, breast cancer is the first cancer of women with 2625 news cases each year [4, 5]. The important risk factors of mammary cancer include an estrogen exposure, age, mutations in tumor suppressor genes (BRCA1 and BRCA2), obesity, and environmental pollutants (polycyclic aromatic hydrocarbons) [6, 7]. Therapeutic management of breast cancer include frequently among other therapies tamoxifen (an antiestrogen drug in hormonal therapy) and chemotherapy's drugs such as doxorubicin (a cytotoxic antibiotic) and paclitaxel (an antimicrotubule agent), both widely used for estrogen-dependent breast cancer and metastatic breast cancer [8, 9]. However, myelosuppression that affect the immune system, serious irreversible cardiotoxicity, and endometrial cancer with thromboembolic events are some undesirable long-term side effects observed with paclitaxel, doxorubicin, and tamoxifen, respectively [8, 10, 11]. Considering these limitations, discovery and development of new chemopreventive drugs against breast cancer with an interesting safety and efficacy to improve breast cancer management and reduce the high cost and pain of patients are an urgent necessity [12].

Breast cancer has been found attenuated by an appreciable amount of natural substances including phytochemicals and dietary substances which affect cell proliferation, cell differentiation, angiogenesis, apoptosis, and a few other cellular transduction pathways [13]. Nevertheless, the continued search for safer and more effective natural agents to improve the efficiency of breast cancer treatment is furthermore a need [14].* Acacia seyal* (Mimosaceae) is a plant of 6–10 m of height with a smooth trunk, alternating leaves, and containing 2–5 yellow glomeruli. It is used in sub-Saharan Africa traditional system medicine to treat many diseases such as infertility, dermatosis, and cancers [15]. This last use brings us to have a great interest in this plant. In addition, no published study on its anticancer activity was found in literature to the best of our knowledge. This work was therefore aimed at evaluating the* in vitro* cytotoxicity of hydroethanolic extract of* A. seyal* in tumoral (MCF-7, MDA-MB-231, SK-MEL-28, SF-295, and 4T1) and nontumoral (NIH-3T3, MRC-5, and HUVEC) cell lines. Further, the protective effects of extract in 7,12- dimethylbenz(a)anthracene- (DMBA-) induced breast cancer in female Wistar rats were also evaluated on mammary tumor incidence, tumor burden, tumor volume, histomorphology, and estrogen target organs. Moreover, safety profile of this extract at the tested doses has also been investigated.

## 2. Materials and Methods

### 2.1. Chemicals and Reagents

Serums and antibiotics were purchased from GIBCO (Grand Island, NY, USA). The 2-[4-(2-hydroxyethyl)piperazin-1-yl]ethane sulfonic acid (HEPES, purity ≥ 99.5%) was purchased from Ludwig Biotecnologia Ltda. (Alvorada, RS, Brazil). DMBA (purity ≥ 95%) was purchased from Sigma-Aldrich (Stanford, Germany). Trypan blue (0.4%), Alamar Blue, and cell culture mediums were purchased from Sigma-Aldrich (St. Louis, MO, USA). Tamoxifen citrate (Mylan®) was purchased from Mylan SAS (Saint-Priest, France). All solutions and buffers were prepared in Ultrapure Milli-Q water.

### 2.2. Plant Material

#### 2.2.1. Collection and Authentication

Stem barks of* Acacia seyal* (Mimosaceae) were collected in Moutourwa (Far-North Region of Cameroon) on 12 March 2015 at about 8:00 am and identified at the Cameroon National Herbarium (CNH) (voucher specimen no. 19223 SFR/Cam) by the botanist Dr. Gilbert Todou. The plant was localized at the geographical coordinates of 10°22′270′′ North and 014°19′686′′ East and 463 m of altitude with a “Garmin” Global Positioning System.

#### 2.2.2. Preparation of the Extract

A total of 2,000 g of well-dried and pulverized stem barks were macerated in 6 liters of distilled water/ethanol mixture (v/v: 30/70) for 2 days at room temperature. Forty grams (2%) of extract was obtained after filtration through a Whatman no. 4 filter paper, evaporation of the ethanol using a rotary evaporator in vacuum under reduced pressure (175 mbar), and lyophilization.

#### 2.2.3. Quantitative Phytochemical Analysis

*(1) Estimation of Total Protein Content.* The quantity of proteins present in* A. seyal* extract was determined by Bradford method [16] using Bovine Serum Albumin (BSA) as standard. Practically,* A. seyal* extract (1 mL) was added to the same volume of Bradford reagent freshly prepared. The absorbance was measured at 595 nm using a UV-VIS 1605 Shimadzu spectrophotometer after incubation for 30 min at darkness.

*(2) Estimation of Total Phenolic Content.* The phenolic compounds were quantified in* A. seyal* extract by the Folin-Ciocalteu methods [17]. Folin-Ciocalteu reagent was added to* Acacia seyal* extract (1 : 10). After 3 min, Na_2_CO_3_ (20%) were added (1 : 2), followed by incubation for 30 min in the dark. The absorbance was measured at 760 nm using a UV-VIS 1605 Shimadzu spectrophotometer. Gallic acid was used as standard during the experimentation.

*(3) Estimation of Flavonoids Content.* The aluminum chloride colorimetric method was done as reported by Chang et al. [18]. A 0.01% quercetin was used to make the calibration curve. The* A. seyal* extract (3 g/100 g solution) was mixed with 2.5% aluminum chloride and methanol (2/1.2/6.8). After incubation at room temperature for 45 min, the absorbance of the reaction mixture was measured at 430 nm using a Shimadzu UV-1700 spectrophotometer. The amount of 2.5% aluminum chloride was substituted by the same amount of methanol in blank. The total flavonoid content was expressed as mg quercetin per gram of* Acacia seyal* extract. Each assay was repeated trice and the results were recorded as mean of the triplicated experiments.

*(4) Estimation of Flavonols Content.* Total flavonols in* A. seyal* extract were estimated as reported by Zhishen et al. [19]. Briefly, sample (standard) and 2% AlCl_3_ ethanol and 50 g/L sodium acetate solutions were added. The absorption at 440 nm was read after 2.5 h of incubation at 20°C. The* A. seyal* extract samples were evaluated at a final concentration of 0.1 mg/mL. Total flavonols content was expressed as mg of quercetin/g of dried extract. Each assay was repeated trice and the results were recorded as mean of the triplicated experiments.

*(5) Evaluation of DPPH (1,1-Diphenyl-2-picryl hydrazyl) Free Radical Scavenging Activity.* The free radical scavenging activity of the* A. seyal* extract was measured in terms of their hydrogen donating or radical scavenging ability using the DPPH radical [20]. For this to be done,* A. seyal *extract at different concentrations (100–300 *μ*g/mL) was introduced into test tubes and 500 *μ*L of the freshly prepared solution of 400 *μ*mol/L DPPH in methanol was then added. The mixture was incubated at 37°C for 30 min in dark and the absorbance was measured at 517 nm using a UV-1605 Shimadzu spectrophotometer. Ferulic acid was used as the positive control. A low absorbance of the reaction mixture indicated high free radical scavenging activity. The DPPH radical scavenging effect was calculated as “inhibition percentage” according to the following formula: percentage of inhibition (%) = {[(*A*0 − *A*1)/*A*0] × 100}, where *A*_0_ is the absorbance of the control reaction and* A*1 is the absorbance in the presence of the sample.

*(6) Evaluation of 2,2-Azino-bis-3-ethylbenzothiazoline-6-sulphonic acid (ABTS) Free Radical Scavenging Assay.* ABTS free radicals scavenging activity was evaluated according to Re et al. [21]. Briefly, 1 mL of ABTS reagent was added to 100 *μ*L of* Acacia seyal* extract at different concentrations (100–300 *μ*g/mL). The mixture was stirred and kept in the dark for 30 min. The absorbance was measured at 734 nm using UV-VIS 1605 Shimadzu spectrophotometer. Ferulic acid was used as the positive control. The ABTS radical scavenging effect was calculated as above.

### 2.3. *In Vitro* Experiment

#### 2.3.1. Cell Lines

Cancer cell lines: MCF-7 (human ER-positive breast adenocarcinoma cells), MDA-MB-231 (human ER-negative breast adenocarcinoma cells), 4T1 (mouse mammary tumor cells), SK-MEL-28 (human melanoma cells), and SF-295 (human glioblastoma cells) and nontumoral cell lines: NIH-3T3 (murine fibroblast cells), HUVEC (human umbilical vein endothelium cells), and MRC-5 (human fetal lung fibroblast cells) were obtained from the Rio de Janeiro Cell Bank (Federal University of Rio de Janeiro, RJ, Brazil).

#### 2.3.2. Cell Culture

Dulbecco's Modified Eagle Medium (DMEM) supplemented with 10% of fetal bovine serum (FBS) was used for the culture of MDA-MB-231, SK-MEL-28, and MRC-5 cells. RPMI-1640 medium supplemented with FBS 10% was used for culture of MCF-7, HUVEC, NIH/3T3, SF-295, and 4T1 cells. All cell cultures were also supplemented with penicillin 100 U/mL, streptomycin 100 *μ*g/mL, and HEPES 10 mM. The conditions were maintained at 37°C in a CO_2_ 5% humidified atmosphere and pH 7.4 for the cell cultures. After every two days, 90% of the supernatant was replaced with fresh medium during the cells passage. Prior to performing all experiments, the trypan blue method and Neubauer chamber were used to assess the number of viable cells and cell count, respectively.

#### 2.3.3. Cell Viability Assay

The Alamar Blue (resazurin) assay was used to test the cytotoxicity of* A. seyal* extract in five tumoral cell lines (MCF-7, MDA-MB-231, SK-MEL-28, SF-295, and 4T1) and three nontumoral cell lines (NIH-3T3, MRC-5, and HUVEC). This assay measures the mitochondrial production as a proof of cell viability [22]. The cytotoxic concentration of* A. seyal* extract which kills 50% of cells (CC_50_) was investigated in all studied cell lines. To achieve this, a 96-well plate was seeded with 100 *μ*L of culture medium having a density of 1 × 10^4^ cells/well and left to adhere overnight. Cells were exposed to* A. seyal* extract at concentrations ranging 25–575 *μ*g/mL after 24 h. The determination of the fluorescent intensity was done using a Perkin Elmer LS55 spectrofluorometer (Becton Dickinson, San Jose, CA) with excitation at 530 nm and emission at 590 nm. The software GraphPad Prism 6.0 (GPW6-242831-RBMZ-03274) was used to calculate CC_50_ which was determined by nonlinear logarithm regression analysis of the logarithm of concentration in function of the normalized response (percentage of cell viability). Each experiment was performed in triplicate and repeated three times.

### 2.4. *In Vivo* Experiment

#### 2.4.1. Animals

Experimental female Wistar rats, aged 25–31 days (39–59 g) used in this study, were obtained from the breeding facility of the Laboratory of Physiology and Natural Products Research, University of Maroua (Cameroon). Animals were housed in plastic cages at room temperature. They had free access to a standard soy-free rat diet and water. The composition of animal diet was corn (36.7%), bone flour (14.5%), wheat (36.6%), fish flour (4.8%), crushed palm kernel (7.3%), sodium chloride (0.3%), and vitamin complex (Olivitazol®- 0.01%). All experiments were conducted in accordance with the principles and procedures of the European Union on Animal Care (CEE Council 86/609) guidelines adopted by the Cameroon Institutional National Ethic Committee, Ministry of Scientific Research and Technology Innovation (Reg. number FWA-IRD 0001954).

#### 2.4.2. DMBA-Induced Breast Tumors in Rats

Seventy-two (72) rats were acclimatized for 18 days and randomly assigned at age of 43–49 days into 6 groups of 12 animals in each. Animals were first treated by intragastric gavage once daily at about 5:00 pm for 7 days. The groups NOR (normal control) and DMBA (negative control) received distilled water as vehicle. Group TAM + DMBA was used as positive control and received tamoxifen citrate (3.3 mg/kg BW). The three remaining groups (AS 50 + DMBA, AS 150 + DMBA, and AS 300 + DMBA) were treated with* A. seyal* extract at the dose of 50, 150, and 300 mg/kg BW, respectively. Then, breast cancer was induced on the 8th day of treatment by a single dose of DMBA (80 mg/kg BW, per os) dissolved in 1 ml of olive oil to all the experimental groups except normal control that received olive oil only. All treatments continued in the same way from the day of cancer induction until 140 days (5 months). Experimental rats were weighed weekly and palpated twice a week to check the development of mammary tumors from the first day of acclimatization until the end of the experiment. Tumorous latency (time of tumor appearance) was recorded. The sacrifice and autopsy either of animals that died during the experiment or of those that became moribund were carried out. At the end of the 5 months of treatment, all survivors were sacrificed after a 12 h overnight nonhydric fasting. Blood was collected on the one hand in anticoagulant (EDTA) tubes for hematological analysis and the other hand in dry tubes and centrifuged at 600 ×g for 15 min for biochemical analysis (transaminase activity, lipid, and creatinine levels). Furthermore, the skin was dissected out to expose mammary tumors and all tumors were removed, counted, and weighed. A 1 mm precision sliding caliper (IGAGING®) was used to measure the size of tumors. Afterward, the tumorous incidence of different groups was recorded and the formula from Faustino-Rocha and collaborators [23] (length × weight × height ×  *π*/6) was used to calculate the tumor volume. Some organs were also removed and weighed. These are estrogen target organs (uterus, vagina, and mammary glands), major metastatic organs of breast cancer (femur, brain, liver, and lungs), and other toxicity organs (spleen, kidneys, and adrenergic glands). Finally, all these organs were fixed in 10% neutral formalin solution for histomorphology.

### 2.5. Histological Analysis

Organs (mammary glands, uterus, vagina, femur, brain, liver, and lung) were dehydrated by a series of ethanol solution and embedded in paraffin blocks before cutting into 5 *μ*m sections and stained with hematoxylin and eosin. Histomorphological changes were determined under an Axioskop 40 microscope connected to a computer where the image was transferred using MRGrab1.0 and Axio Vision 3.1 software (Zeiss, Hallbergmoos, Germany). Atlas and histologic classification of tumors of rat mammary gland from J. Russo and I. H. Russo [24] were used in this study.

### 2.6. Biochemical and Hematological Analysis

Regarding biochemical analysis, triglycerides (TG), total cholesterol (TC), high-density lipoprotein (HDL-C), alanine transaminase (ALT) activity, aspartate transaminase (AST) activity, and creatinine level were measured using reagent kits from Fortress Diagnostics Limited (Muckamore, UK). LDL cholesterol was estimated from total cholesterol, HDL cholesterol, and triglycerides by using the formula of Friedewald et al. [25]. Atherogenic index (AI) was calculated as a ratio of total cholesterol to HDL cholesterol. In addition, atherogenic index of plasma (AIP) related to the particle size of lipoproteins was calculated as the logarithm of triglycerides/HDL cholesterol ratio [26].

Different hematological parameters were evaluated using a Mindray BC-2800 Auto Hematology Analyzer from Shenzhen Mindray Bio-Medical Electronics Co., Ltd. These are white blood cell (WBC) count, lymphocytes, monocytes, granulocytes, red blood cell (RBC) count, hematocrit, hemoglobin, mean corpuscular volume (MCV), mean corpuscular hemoglobin (MCH), mean corpuscular hemoglobin concentration (MCHC), and platelets.

### 2.7. Statistical Analysis

Results are presented as means ± standard error of mean (SEM) in triplicate from three independent* in vitro* experiments and for each* in vivo *experimental group. Statistical analysis of data with GraphPad Prism software version 6.00 (GPW6-242831-RBMZ-03274) were realised using the one-way analysis of variance (ANOVA) followed by Dunnett's post hoc test for multiple comparisons. Statistical significance of differences was considered at a *p* value < 0.05.

## 3. Results

### 3.1. Preliminary Phytochemical Analysis

The quantitative phytochemical analyses revealed that* A. seyal* ethanolic extract contains by g of dried weight 88.15 ± 3.23 mg eq Bovine Serum Albumin, 90.16 ± 1.28 mg eq gallic acid, 46.79 ± 14.18 mg eq quercetin, and 16.31 ± 5.72 mg eq quercetin of total proteins, total phenols, flavonoids, and flavonols, respectively (Table 1). Moreover, it can be observed that the efficacy concentrations of* A. seyal* extract which results in 50% of scavenging (EC_50_) are 205.4 *μ*g/mL (DPPH) and 202.3 *μ*g/mL (ABTS).

### 3.2. Cytotoxicity of* A. seyal* Extract

Table 2 depicts the cytotoxicity of* A. seyal* extract on five cancer cell lines (MCF-7, MDA-MB-231, 4T1, SK-MEL-28, and SF-295) and three nontumoral cell lines (NIH-3T3, HUVEC, and MRC-5). After 24 h of incubation* A. seyal* extract was observed a significant cytotoxicity on different cancer cell lines with a more pronounced effect in estrogen receptor-positive cells (MCF-7, CC_50_ = 100 *μ*g/mL), mouse mammary tumor cells (4T1, CC_50_ = 25 *μ*g/mL), and human glioblastoma cells (SF-295, CC_50_ = 100 *μ*g/mL). Concerning nontumoral cell lines, except murine fibroblast cells (NIH/3T3) where the CC_50_ was also pronounced (36 *μ*g/mL), the cytotoxicity was less important in other nontumoral cell lines [HUVEC (CC_50_ = 204 *μ*g/mL) and MRC-5 (CC_50_ = 575 *μ*g/mL)].

### 3.3. Protective Effects of* A. seyal* Extract on Mammary Tumors in Rats

#### 3.3.1. Effects on Body Weight and Survival Rate

**Figure 1:**
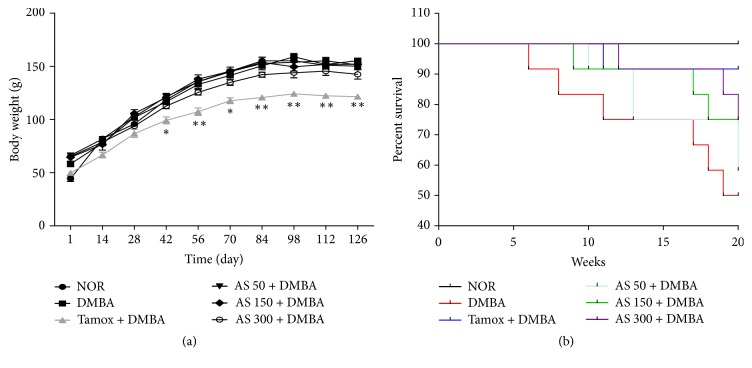
Body weight evolution (a) and Kaplan-Meir survival curve (b) after 20 weeks of treatment. NOR = normal control treated with water; DMBA = DMBA control treated with water; AS + DMBA = animals treated with the* A. seyal *hydroethanolic aqueous extract at the doses of 50, 150, and 300 mg/kg. TAM + DMBA = animals treated with tamoxifen (3.3 mg/kg); all groups excepting the normal group (NOR) received an intragastric dose of DMBA at the dose of 80 mg/kg. Data are represented as mean ± SEM (*n* = 12). Data are represented as mean ± SEM (*n* = 10). ^*∗*^*p* < 0.05, ^*∗∗*^*p* < 0.01 as compared to negative control.

The effects of* A. seyal* extract were evaluated after 20 weeks of treatment on body weight and survival rate (Figure 1). Any treatment during the entire study except tamoxifen did not significantly affect the body weight of animals among the experimental groups. Animals treated with tamoxifen presented a significant (*p* < 0.01) lower body weight as compared to the normal control group (Figure 1(a)).

In the course of 20 weeks of treatment, the highest death rate was recorded in the DMBA group (50%), followed by low dose (50 mg/kg) of* A. seyal* extract (40%) and tamoxifen group (10%) (Figure 1(b)).

#### 3.3.2. Effects on Mammary Tumors

Table 3 presents data related to chemopreventive activity of* A. seyal* extract on mammary tumor incidence, total tumor burden, and average tumor weight after 20 weeks of treatment. At the end of the experiment, the animals from the normal group presented no tumor while the animals of the group that received only the carcinogen (DMBA) presented 91.66% of mammary tumors. As expected, animals treated with tamoxifen showed a significant (*p* < 0.001) reduction in tumor incidence (25%) and an average tumor weight of 82.9% as compared to DMBA control animals. Interestingly,* A. seyal* extract (150 and 300 mg/kg) significantly (*p* < 0.01 and *p* < 0.001 respectively) reduced in a dose-responsive manner the tumor incidence (41.66% and 25%) and an average tumor weight of 62.1% and 65.8% as compared to DMBA group.

**Figure 2:**
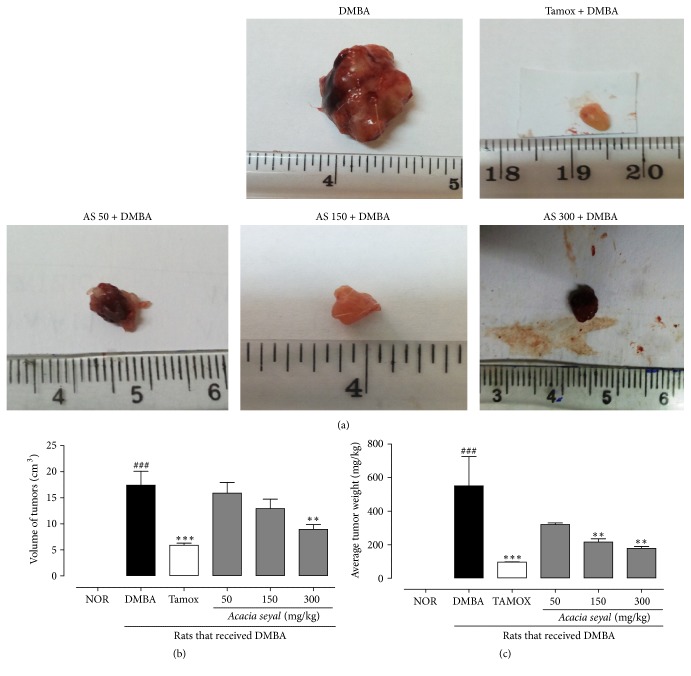
Effects of* A. seyal* extract on mammary gland tumors (a), volume of tumors (b), and tumors relative weight (c) after 20 weeks of treatment. NOR = normal control treated with water; DMBA = DMBA control treated with water; AS + DMBA = animals treated with the* A. seyal *hydroethanolic aqueous extract at the doses of 50, 150, and 300 mg/kg. TAM + DMBA = animals treated with tamoxifen (3.3 mg/kg); all groups excepting the normal group (NOR) received an intragastric dose of DMBA at the dose of 80 mg/kg. Data are represented as mean ± SEM (*n* = 10). Data are represented as mean ± SEM (*n* = 12). Data are represented as mean ± SEM (*n* = 10). ^*∗∗*^*p* < 0.01, ^*∗∗∗*^*p* < 0.001 as compared to negative control. ^###^*p* < 0.001 as compared to normal.

Figure 2 presents data related to protective effect of* A. seyal* extract on photographs, mammary tumor volumes, and average tumor weights after 20 weeks of treatment. At the end of the experiment, no mammary tumors were observed in the rats of the normal control group while most of the mammary tumors of animals treated with DMBA were large (≈500 mg/kg). Animals treated with tamoxifen and* A. seyal* extract at all tested doses (50, 150, and 300 mg/kg) presented a striking reduction of the size of mammary tumors as compared to DMBA control group (Figure 2(a)). In this study, tamoxifen significantly (*p* < 0.001) decreased mammary tumor volume and average tumor weight as compared to DMBA control group (Figures 2(b)-2(c)). Interestingly, the* A. seyal* extract in a dose-responsive manner exhibited such effects significantly (*p* < 0.01) decreasing the average of tumor weight at medium dose (150 mg/kg) and high (300 mg/kg) dose, while the volume of tumors significantly (*p* < 0.01) decreased only with the high dose (300 mg/kg) as compared to DMBA animals. A decrease in the average tumor weight was also observed at the low dose (50 mg/kg) but it did not reach the level of statistical significance.

#### 3.3.3. Histomorphological Analysis of Estrogen Target Organs

**Figure 3:**
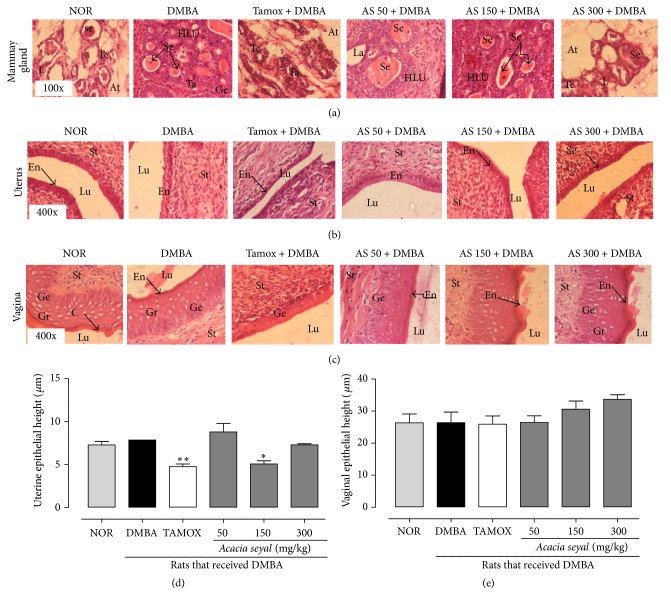
Effects of* A. seyal* extract on microphotographs H&E 400× of mammary glands (A), uterine (B), and vagina (C) and graphic representations of uterine (D) and vagina (E) height after 20 weeks of treatment. NOR = normal control treated with water; DMBA = DMBA control treated with water; AS + DMBA = animals treated with the* A. seyal* hydroethanolic aqueous extract at the doses of 50, 150, and 300 mg/kg. TAM + DMBA = animals treated with tamoxifen (3.3 mg/kg); all groups except the normal group (NOR) received an intragastric dose of DMBA at the dose of 80 mg/kg. Data are represented as mean ± SEM (*n* = 12). Data are represented as mean ± SEM (*n* = 12). Data are represented as mean ± SEM (*n* = 10). ^*∗*^*p* < 0.05, ^*∗∗*^*p* < 0.01 as compared to negative control. La = lumen of alveoli; At = adipose tissue; Se = eosinophil secretion; L = lobular; HLU = hyperplastic lobular unit; St = stroma; En = endometrium; Lu = lumen of uterine; Ge = germinal epithelium.

The histomorphology of the mammary glands of animals from the normal group present normal acini surrounded by a small amount of fibrous conjunctive tissue and an important eosinophil secretion (Figure 3(a)). Twenty weeks after DMBA administration, in situ carcinoma of the mammary gland was detected, characterized by severe hyperplasia of mammary lobules and the dilated ducts filled with tumoral cells with a diminution of the conjunctive tissue. The histoarchitecture of the mammary gland from animals treated with tamoxifen presented a quasinormal structure; no sign of hyperplasia was observed, with small lobules and well differentiated acini. Animals treated with* A. seyal* extract presented hyperplasia, which was dose-dependent in an inverse proportional manner, AS 300 + DMBA having quasinormal histoarchitecture with low cellular proliferation and low ductal dilation.

The tamoxifen significantly (*p* < 0.01) decreased the uterine epithelial height (Figure 3(b)) but not the vaginal epithelial height (Figure 3(c)).* A. seyal* also induced a significant (*p* < 0.05) decrease in uterine epithelial height at medium dose (150 mg/kg), but a dose-dependent increase in vaginal epithelial height, though not statistically significant.

#### 3.3.4. Effects of* A. seyal* Treatment on Relative Organ Weights

The relative weights of various organs following 20 weeks of treatment with* A. seyal* extract are displayed in Table 4. Between the normal and DMBA control groups, no significant difference in the weight of various organs was observed except for the adrenal glands and ovaries weights which presented significant decreases in DMBA control with *p* < 0.05 and *p* < 0.001, respectively. Treatment of rats with tamoxifen significantly (*p* < 0.001) decreased uterine wet weight and increased brain weight as compared to DMBA control group. Following treatment with* A. seyal* extract, the group AS 50 + DMBA showed increase in the lungs weight (*p* < 0.05), while AS 150 + DMBA showed increase in liver weight. It is worth noting that there was no significant difference in all observed organ weights between AS 300 + DMBA and either normal or DMBA control groups at the end of the experiment. Also, there was no significant difference in the femur (one of the major metastatic sites) weight in all groups.

#### 3.3.5. Effects on Various Toxicological Parameters

Table 5 depicts the effects of* A. seyal* extract on some toxicological biochemical parameters. Rats that received only DMBA presented a significant (*p* < 0.001) increase in the transaminases (ALT and AST) activities as compared to normal rats.* A. seyal* extract at low dose (50 mg/kg) showed a significant increase in both enzymes activities, ALT (*p* < 0.001) and AST (*p* < 0.01). The medium dose of* A. seyal* extract (150 mg/kg) also showed significant increase in ALT activity (*p* < 0.01) while the high dose (300 mg/kg) showed significant decrease in AST activity (*p* < 0.001) as compared to DMBA rats. On fasting lipid levels, a statistically significant (*p* < 0.01) reduction in the LDL cholesterol level was found with tamoxifen and* A. seyal* extract (150 mg/kg) as compared to DMBA animals. It was also observed with* A. seyal* extract at high dose (300 mg/kg) a significant decrease in the LDL cholesterol (*p* < 0.01) and HDL cholesterol (*p* < 0.05) levels as compared to DMBA control group. Concerning creatinine content in blood, an increase was noted in DMBA-treated rats as compared to normal group, but no significant changes were observed between animals treated with tamoxifen and* A. seyal* extract at all tested doses.

No statistical significance was observed between normal control and DMBA control groups in all measured parameters except for a significant (*p* < 0.001) increase in white blood count (WBC) in the latter compared to the former (Table 6). Tamoxifen-treated animals showed a significant (*p* < 0.05) decrease in WBC, platelets, and red blood count (RBC) as compared to DMBA and normal groups.* A. seyal* extract at the dose of 50 and 300 mg/kg (*p* < 0.05 and *p* < 0.01 respectively) significantly decreased monocytes number as compared to DMBA group. Animals treated with* A. seyal* extract (300 mg/kg) also induced a significant decrease in RBC (*p* < 0.01), hematocrit (*p* < 0.001), mean corpuscular volume (MCV) (*p* < 0.05), platelets (*p* < 0.05), and hemoglobin (*p* < 0.01) as compared to DMBA group.

#### 3.3.6. Effects on Metastatic Organs

**Figure 4:**
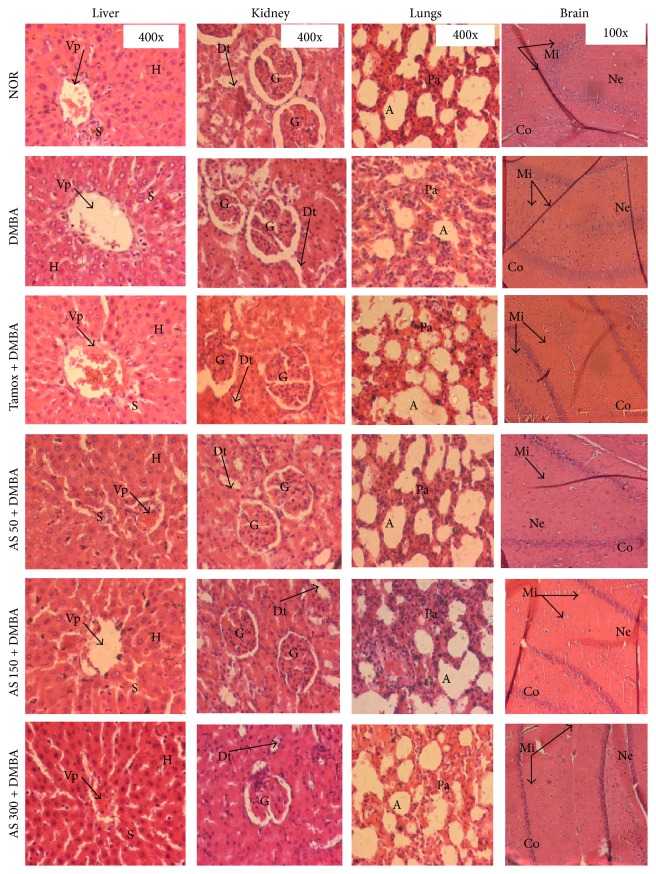
Effects of* A. seyal* extract on microphotographs H&E 400x of liver, kidney, lung, and brain after 20 weeks of treatment. NOR = normal control treated with water; DMBA = DMBA control treated with water; AS + DMBA = animals treated with the* A. seyal *hydroethanolic aqueous extract at the doses of 50, 150, and 300 mg/kg. TAM + DMBA = animals treated with tamoxifen (3.3 mg/kg); all groups excepting the normal group (NOR) received an intragastric dose of DMBA at the dose of 80 mg/kg. Data are represented as mean ± SEM (*n* = 10). Data are represented as mean ± SEM (*n* = 12). Data are represented as mean ± SEM (*n* = 10). Vp = portal vein; H = hepatocyte; S = sinusoids; A = alveoli; Ba = aveolar bag; G = glomerulus; Dt = distal tube; Pt = proximal tube; Mi = microglia; Ne = neuron; Co = cortex.

The liver, lungs, brain, and bone are known as major metastatic sites of breast cancer. In addition, liver, lungs, and kidney are the primary organs of toxicity. These organs have therefore been explored histologically (Figure 4). At the end of the experiment, no metastasis and changes were noted in the histoarchitecture of these organs except for the liver of animals of the DMBA control group, which presented an increase in the number of pyknotic nuclei.

## 4. Discussion

Mortality rate due to breast cancer remains high, though much progress is being made in producing safer drugs [27]. Novel molecules are continuously being identified and developed from medicinal plants, which in general are less toxic and have been presented effective results [28]. It has been proven from extensive* in vitro *and* in vivo *studies that botanical extracts as well as isolated phytoconstituents derived from plants especially of desert and semidesert habitats exert potent anticancer activities [29, 30]. To contribute to the quest for safer anticancer molecules the* in vitro* and* in vivo* properties of the hydroalcoholic stem bark extract of* Acacia seyal* were explored.

Regarding our* in vitro* antitumoral assays, one of our most important results shows that* A. seyal* extract demonstrated cytotoxic activity in all tested cell lines with CC_50_ values ranging 25–265 *μ*g/mL for the tumoral cell lines as opposed to 36–575 *μ*g/mL for nontumoral cell lines. The higher activity of the extract in estrogen receptor-positive cells MCF-7 with CC_50_ of 100 *μ*g/mL than in estrogen receptor-negative cell MDA-MB-231 with CC_50_ value of 265 *μ*g/mL is of particular interest. This result suggests that estrogen receptors are implicated in the mechanism by which* A. seyal* extract induces cytotoxicity. However, it would not act solely through estrogen receptors since the murine fibroblast cells (NIH/3T3) were also sensitive to* A. seyal* extract with a CC_50_ value of 36 *μ*g/mL. Apart from this exception, tumor cells generally appeared more sensitive to* A. seyal* extract than normal cells. This suggests that the extract has a high selectivity for these tumor cells than for nontumoral cells, which is of interest in the quest for alternative breast cancer treatment. These effects are thought to be due to the presence of anticancer components in the extract. As displayed in the preliminary phytochemical analysis,* A. seyal* extract contains flavonoids, particularly flavonols, which might account for its cytotoxicity because quercetin the most abundant flavonol in the plants has been described as cytotoxic* in vitro* [31–33].

For* in vivo* experiment, an animal model for breast cancer was used to evaluate the protective effects of* A. seyal* extract. 7,12-Dimethylbenz(a)anthracene (DMBA), which is a polycyclic aromatic hydrocarbon, is also an environmental carcinogen frequently used to induce cancer in experimental animals because the breast tumors produced closely mimic those of human breast cancer [24]. Individuals constantly come in contact with low doses of this substance by consumption of meat exposed to very high temperatures and by exposure to fossil fuels that undergo incomplete combustion and cigarette smoke [34, 35]. In this study, DMBA was administered at a single dose of 80 mg/kg BW by intragastric gavage to Wistar rats aged 50–56 days to induce mammary tumors [36, 37]. Due to the active proliferation of the terminal ducts in breast tissue for rats in this range of age, they become very susceptible to carcinogens and tumor development [38]. Experimental animals that received only DMBA developed large tumors after 20 weeks of study, whereas normal control animals did not exhibit tumors. This result is in agreement with studies of Bishayee and Mandal [39] and Bishayee and collaborators [40] that found similar results with DMBA in female Sprague-Dawley rats. The significant reduction in tumor volume and average tumor weight observed with* A. seyal* extract in a dose-dependent manner such as tamoxifen in this study suggests protective effects of this extract on the mammary tumorigenesis. These effects could be explained by the ability of phytoconstituents of* A seyal* extract to kill cancer cells, as observed* in vitro *in this study. Compounds found in* A. seyal* extract are known to be anticancerous. Of note, breast tissue is a well-known target of flavonols, which could account for the observed cancer protective potential of* A. seyal* because these compounds are proapoptotic and endowed with cell cycle arrest property [31].

Plant extracts consist of a mosaic of compounds displaying more than one mode of action on several targets. The significant (*p* < 0.05) decrease in uterine epithelial height observed with* A. seyal* extract (150 mg/kg) on estrogen target organs could suggest an antiestrogenic effect of this extract, which would be interesting. Flavonoids form the largest group of natural phenolic compounds and possess excellent estrogen-like effects. These later could account for the antiestrogenic activity observed with* A. seyal* extract on uterus in this study. In fact, at 100- to 1000-fold concentrations, phytoestrogens can enter in competition with endogen estrogens for ERs [41]. Knowing that antiestrogenic effects are a needed effect on estrogen-dependent tumors, we can hypothesize that flavonoids, mainly flavonols detected in* A. seyal* extract, might bind* in vivo* by an estrogen-dependent pathway to inhibit the proliferation of tumor cells. Hence,* A. seyal* extract in addition to its antimammary tumor effects might prevent estrogen-dependent endometrial cancer. Results obtained on histopathological examination of the mammary gland sections are in line with those observed in tumor volume and average tumor weight. The in situ carcinoma noted in DMBA control animals as compared to normal group that exhibited a quite-normal histological sections is in accordance with several studies, which showed that DMBA alters the normal process of mammary gland differentiation of terminal ducts to alveoli and lobules [30, 42–44]. However, tamoxifen and* A. seyal* extract almost at the dose of 300 mg/kg protected mammary glands against DMBA-induced histopathological alterations.

Regarding toxicological profile, relative weight of organs is an indicator of toxic effects of tested substances [45]. The significant decrease in adrenal glands (*p* < 0.05) and ovaries (*p* < 0.001) weights observed in DMBA control group could be explained by toxic effects of DMBA which is able to induce in rats hemorrhagic injuries (apoplexy) in different organs such as adrenals [46]. Nevertheless, variations of lungs (increase) and liver (decrease) weights noted with* A. seyal* extract, respectively, at the dose of 50 and 150 mg/kg could not be linked with a toxic effect, since the higher dose (300 mg/kg) did not induce such effect.

## 5. Conclusion

In conclusion, we have demonstrated here that the ethanolic extract of* Acacia seyal* has cytotoxic effects against a variety of tumor cells. It protected rats against DMBA-induced breast hyperplasia as well as breast tumor incidence, with an optimal effect at the dose of 300 mg/kg. Furthermore, no evidence of toxicity was observed with this extract at the tested doses during the 20 weeks of treatment. Overall,* A. seyal* is a plant that has potential* in vitro* and* in vivo* anticancer effects, suggesting that it is a promising candidate for the preparation of an improved traditional medicine for cancer. The next step is to make a deep phytochemical investigation to point out the bioactive components of* A. seyal* extract and to elucidate its underlying mechanisms.

## Figures and Tables

**Table 1:** Quantitative phytochemical analyses and scavenging free radical activities of *A. seyal* extract.

Concentration in *A. seyal *extract of selected phytochemicals			
Total proteins	88 ± 3 mg eq bovine serum albumin		
Total phenols	90 ± 1 mg gallic acid		
Flavonoids	47 ± 14 mg eq quercetin		
Flavonols	16 ± 6 mg eq quercetin		

Scavenging free radical activities of *A. seyal*			

		EC_50_ (*µ*g/mL)	
		DPPH	ABTS

Control (ferulic acid)		4.3	3.7
*A. seyal*		205.4	202.3

EC_50_ = concentration of *Acacia seyal* extract which results in 50% of scavenging.

**Table 2:** Comparative CC_50_ values of hydro-ethanolic extract of *Acacia seyal* in tumoral and nontumoral cell lines.

CC_50_ (*µ*g/mL)			
(a) Tumor cell lines		(b) Nontumor cell lines	
MCF-7	100	HUVEC	204
MDA-MB-231	265	MRC-5	575
4T1	25	NIH-3T3	36
SK-MEL-28	132		
SF-295	100		
	124.4 ± 35.15		271.66 ± 78.01

CC_50_ = concentration of *Acacia seyal* extract which results in 50% of cell viability. No statistical significance was found between both groups.

**Table 3:** Breast cancer chemopreventive activity of *A. seyal* extract after 20 weeks of treatment.

Items	NOR	DMBA	Tamox + DMBA	AS 50 + DMBA	AS 150 + DMBA	AS 300 + DMBA
Number of rats with tumors/total rats	0/12	11/12	3/12	7/12	5/12	3/12
Tumor incidence (%)	0	91.66^###^	25^*∗∗∗*^	58.33	41.66^*∗∗*^	25^*∗∗∗*^
Average tumor weight (g/kg)	-	569.04 ± 183.8	96.75 ± 1.96^*∗∗∗*^	320.07 ± 15.29	216.18 ± 25.94^*∗∗*^	194.43 ± 15.81^*∗∗*^
% inhibition related to tumor weight	-	-	82.9	43.7	62.1	65.8
Total tumor burden (g)	0	5.51	0.97	3.2	2.16	1.78
% Inhibition related to tumor burden	-	-	82.39	41.92	60.79	67.69

NOR = normal control treated with 2% ethanol; DMBA = DMBA control treated with 2% ethanol; AS + DMBA = animals treated with the *Acacia seyal *hydroethanolic extract at the doses of 50 and 200 mg/kg. TAM + DMBA = animals treated with tamoxifen (3.3 mg/kg); all groups excepting the normal group (NOR) received an intragastric dose of DMBA at the dose of 80 mg/kg. Data are represented as mean ± SEM (*n* = 12). ^*∗∗*^*p* < 0.01 and ^*∗∗∗*^*p* < 0.001 as compared to negative control; ^###^*p* < 0.001 as compared to normal control.

**Table 4:** Effects of *A. seyal *extract on relative weight of various organs after 20 weeks of treatment.

Organs	NOR	DMBA	Tamox + DMBA	AS 50 + DMBA	AS 150 + DMBA	AS 300 + DMBA
Uterus	4817.11 ± 435.16	2981.94 ± 284.88	864.61 ± 55.67^*∗∗∗*^	2879.16 ± 221.22	2410.66 ± 164.74	2261.11 ± 272.49
Liver	25493.86 ± 235.80	30280.67 ± 1373.71	29268.06 ± 963.84	29091.83 ± 475.24	25993.99 ± 1058.97^*∗∗*^	25903.52 ± 512.22
Lungs	8062.07 ± 406.91	8325.98 ± 1188.25	9702.22 ± 1069.33	11214.08 ± 410.51^*∗*^	8720.07 ± 434.87	9953.65 ± 405.30
Spleen	3068.06 ± 112.22	3103.49 ± 380.63	2751.56 ± 192.04	3028.08 ± 299.37	2946.84 ± 251.59	3458.95 ± 149.62
Adrenals	732.68 ± 112.99	477.00 ± 69.19^#^	453.48 ± 16.35	495.42 ± 19.69	347.06 ± 28.11	418.99 ± 15.23
Kidneys	5890.92 ± 94.92	6039.96 ± 114.75	6291.32 ± 208.36	6210.50 ± 246.98	5611.14 ± 273.64	5659.22 ± 102.20
Femur	2517.11 ± 34.79	2428.19 ± 81.83	2669.94 ± 124.11	2738.12 ± 84.96	2500.99 ± 48.11	2589.30 ± 143.58
Brain	10318.64 ± 110.21	9644.14 ± 931.75	12914.09 ± 205.89^*∗∗∗*^	10315.18 ± 185.69	9599.63 ± 253.78	10905.24 ± 197.89
Ovaries	2237.27 ± 404.69	686.05 ± 71.22^###^	548.79 ± 36.90	795.58 ± 25.97	662.74 ± 31.06	593.18 ± 26.82

NOR = normal control treated with 2% ethanol; DMBA = DMBA control treated with water; AS + DMBA = animals treated with the *Acacia seyal *hydroethanolic extract at the doses of 50, 150 and 300 mg/kg. TAM + DMBA = animals treated with tamoxifen (3.3 mg/kg); all groups excepting the normal group (NOR) received an intragastric dose of DMBA at the dose of 80 mg/kg. Data are represented as mean ± SEM (*n* = 12). ^*∗*^*p* < 0.05, ^*∗∗*^*p* < 0.01 and ^*∗∗∗*^*p* < 0.001 as compared to negative control; ^#^*p* < 0.05 and ^###^*p* < 0.001 as compared to normal control.

**Table 5:** Effects of *A. seyal *hydroethanolic extract on some toxicological biochemical parameters after 20 weeks of treatment.

Item	NOR	DMBA	TAM + DMBA	AS 50 + DMBA	AS 150 + DMBA	AS 300 + DMBA
*Transaminases*						
ALT	19 ± 1.35	44.8 ± 2.93^###^	51.6 ± 2.18	59.66 ± 0.99^*∗∗∗*^	55.8 ± 2.01^*∗∗*^	41.33 ± 3.02
AST	16.6 ± 1.53	30.4 ± 2.51^###^	31.16 ± 3.89	41.33 ± 0.43^*∗∗*^	34.4 ± 1.77	15.8 ± 0.81^*∗∗∗*^

*Fasting Lipid levels*						
Total cholesterol (g/L)	91.8 ± 2.17	84.4 ± 4.48	80.83 ± 5.47	86 ± 4.03	82.4 ± 1.89	70.66 ± 0.93
TG (mg/dL)	116.2 ± 5.04	109.16 ± 4.94	119 ± 8.98	112.66 ± 6.19	97.8 ± 1.53	91.5 ± 0.33
Chol-HDL (g/L)	48.8 ± 0.61	52.2 ± 9.04	39 ± 0.5	20.53 ± 7.8	41.6 ± 0.36	36 ± 0.22^*∗*^
Chol-LDL (g/L)	21.56 ± 2.83	35.23 ± 4.59	20 ± 4.28^*∗*^	22.8 ± 2.98	18.9 ± 2.67^*∗*^	15.66 ± 1.05^*∗∗*^
Chol-T/Chol-HDL	1.88 ± 0.053	1.96 ± 0.35	2.06 ± 0.11	2.11 ± 0.11	1.98 ± 0.05	1.96 ± 0.03
log_10_ (TG/Chol-HDL)	0.37 ± 0.02	0.36 ± 0.07	0.47 ± 0.03	0.44 ± 0.03	0.37 ± 0.01	0.41 ± 0.01

*Creatine (g/L)*	1,12 ± 0,03	1,05 ± 0,10^###^	1,41 ± 0,07	1,13 ± 0,04	1,08 ± 0,06	1,13 ± 0,04

NOR = normal control treated with water; DMBA = DMBA control treated with 2% ethanol; TAM + DMBA = positive control treated with tamoxifen (3.3 mg/kg); AS + DMBA = animals treated with the *A. seyal* extract (v/v: 1/1) at the doses of 50, 150, and 300 mg/kg. All groups except of normal group (NOR) received an intragastric dose of DMBA at the dose of 80 m/kg. Data are represented as mean ± SEM (*n* = 12). ^*∗*^*p* < 0.05, ^*∗∗*^*p* < 0.01 and ^*∗∗∗*^*p* < 0.001 as compared to negative control; ^###^*p* < 0.001 as compared to normal control.

**Table 6:** Effects of *A. seyal *hydroethanolic extract on hematological parameters after 20 weeks of treatment.

Items	NOR	DMBA	Tamox + DMBA	AS 50 + DMBA	AS 150 + DMBA	AS 300 + DMBA
WBC (×10^3^*µ*L^−1^)	3,3 ± 0,2	6,04 ± 0,47^##^	3,5 ± 0,40^*∗*^	6,9 ± 0,52	4,32 ± 0,46	4,46 ± 0,71
Lymphocytes (%)	63,74 ± 1,50	61,6 ± 1,17	61,8 ± 1,17	67,66 ± 0,71	62,08 ± 2,51	63,66 ± 0,68
Monocytes (%)	14,62 ± 0,52	16,8 ± 0,75	19,21 ± 0,61	12 ± 0,67^*∗*^	15,56 ± 1,84	11,33 ± 0,56^*∗∗*^
Granulocytes (%)	21,64 ± 1,06	21,6 ± 1,13	18,78 ± 1,21	20,33 ± 0,25	22,36 ± 0,84	25 ± 0,22
RBC (×10^3^ *µ*L^−1^)	7,66 ± 0,21	7,22 ± 0,11	6,81 ± 0,55^#^	6,88 ± 0,18	7,13 ± 0,08	5,14 ± 0,50^*∗∗*^
Hematocrit (%)	48,46 ± 1,20	45,76 ± 1,02	45,51 ± 2,31	43,96 ± 1,31	44,5 ± 0,8	30,1 ± 2,93^*∗∗∗*^
MCV (fL)	63,86 ± 0,31	63,34 ± 0,67	64,85 ± 2,37	63,9 ± 0,26	62,5 ± 0,64	58,83 ± 0,27^*∗*^
Platelets (×10^3^ *µ*L^−1^)	479,6 ± 47,87	435,2 ± 23,21	237,5 ± 36,43^*∗*^	383 ± 54,14	478 ± 17,32	259,66 ± 24,62^*∗*^
MCH (pg)	18 ± 0,14	18,04 ± 0,16	18,83 ± 1,10	17,6 ± 0,12	17,9 ± 0,17	17,13 ± 0,12
Hemoglobin (g/dL)	137 ± 3,13	129,2 ± 2,04	131,33 ± 4,77	121,66 ± 4,00	128,2 ± 1,90	88,33 ± 8,83^*∗∗*^
MCHC (g/dL)	282,4 ± 1,38	282,4 ± 2,77	290 ± 5,63	275,66 ± 1,64	287,4 ± 1,62	291,66 ± 1,12

NOR = normal control treated with water; DMBA = DMBA control treated with water; AS + DMBA = animals treated with the *A. seyal *hydroethanolic aqueous extract at the doses of 50, 150 and 300 mg/kg. TAM + DMBA = animals treated with tamoxifen (3.3 mg/kg); all groups excepting the normal group (NOR) received an intragastric dose of DMBA at the dose of 80 mg/kg. Data are represented as mean ± SEM (*n* = 12). ^*∗*^*p* < 0.05 and ^*∗∗*^*p* < 0.01 as compared to negative control; ^#^*p* < 0.05 as compared to normal control. ^*∗∗∗*^*p* < 0.001 as compared to the negative control; ^##^*p* < 0.01 as compared to normal control.

## Abbreviations

AS: Ethanolic extract of* A. seyal*
ALT: Alanine transaminase
AST: Aspartate transaminase
BW: Body weight
CC_50_: Cytotoxic concentration for 50% of the cells
DMEM: Dulbecco's Modified Eagle Medium
DMBA: 7,12-Dimethylbenz(a)anththracene
DMSO: Dimethylsulfoxide
ER: Estrogen receptor
FBS: Fetal bovine serum
FCS: Fetal calf serum
HEPES: 4-(2-Hydroxyethyl)-1-piperazineethanesulfonic acid
Hb: Hemoglobin
Ht: Hematocrit
MDA: Malondialdehyde
MCH: Mean corpuscular hemoglobin
MCHC: Mean corpuscular hemoglobin concentration
MCV: Mean corpuscular volume
MeOH: Methanol
NHC: National Herbarium of Cameroon
NOR: Normal control
RBC: Red blood cell
RPMI: Roswell Park Memorial Institute medium
ROS: Reactive oxygen species
SEM: Standard error of mean
TAM: Tamoxifen.
